# Application of Homozygosity Haplotype Analysis to Genetic Mapping with High-Density SNP Genotype Data

**DOI:** 10.1371/journal.pone.0005280

**Published:** 2009-04-28

**Authors:** Haiyan Jiang, Andrew Orr, Duane L. Guernsey, Johane Robitaille, Géraldine Asselin, Mark E. Samuels, Marie-Pierre Dubé

**Affiliations:** 1 Department of Pathology, Dalhousie University, Halifax, Nova Scotia, Canada; 2 Department of Ophthalmology and Visual Sciences, Dalhousie University, Halifax, Nova Scotia, Canada; 3 Department of Pediatrics, Dalhousie University, Halifax, Nova Scotia, Canada; 4 Montreal Heart Institute, Montreal, Quebec, Canada; 5 Department of Medicine, Université de Montréal, Montreal, Quebec, Canada; 6 Centre de Recherche du CHU Ste-Justine, Montreal, Quebec, Canada; Ohio State University Medical Center, United States of America

## Abstract

**Background:**

In families segregating a monogenic genetic disorder with a single disease gene introduction, patients share a mutation-carrying chromosomal interval with identity-by-descent (IBD). Such a shared chromosomal interval or haplotype, surrounding the actual pathogenic mutation, is typically detected and defined by multipoint linkage and phased haplotype analysis using microsatellite or SNP genotype data. High-density SNP genotype data presents a computational challenge for conventional genetic analyses. A novel non-parametric method termed Homozygosity Haplotype (HH) was recently proposed for the genome-wide search of the autosomal segments shared among patients using high density SNP genotype data.

**Methodology/Principal Findings:**

The applicability and the effectiveness of HH in identifying the potential linkage of disease causative gene with high-density SNP genotype data were studied with a series of monogenic disorders ascertained in eastern Canadian populations. The HH approach was validated using the genotypes of patients from a family affected with a rare autosomal dominant disease Schnyder crystalline corneal dystrophy. HH accurately detected the ∼1 Mb genomic interval encompassing the causative gene UBIAD1 using the genotypes of only four affected subjects. The successful application of HH to identify the potential linkage for a family with pericentral retinal disorder indicates that HH can be applied to perform family-based association analysis by treating affected and unaffected family members as cases and controls respectively. A new strategy for the genome-wide screening of known causative genes or loci with HH was proposed, as shown the applications to a myoclonus dystonia and a renal failure cohort.

**Conclusions/Significance:**

Our study of the HH approach demonstrates that HH is very efficient and effective in identifying potential disease linked region. HH has the potential to be used as an efficient alternative approach to sequencing or microsatellite-based fine mapping for screening the known causative genes in genetic disease study.

## Introduction

SNP genotyping technology is developing very rapidly. Phase II of the International HapMap Project has characterized over 3.1 million single nucleotide polymorphisms (SNPs) with the resulting SNP density of approximately one per kilobase [1]. High-density SNP genotyping has gradually become a dominant data source in molecular genetic and clinical laboratories. Illumina Infinium Human1M-Duo and Human610-Quad Beadchips and Affymetrix 6.0 GeneChips are currently amongst the most advanced genotyping platforms. The rapid advances in high-throughput genotyping technologies require efficient methods which can fully employ the profound information provided by the high-density SNP genotype data.

High-density SNP genotype data presents a computational challenge for genetic analyses. Disease gene mapping methods, including linkage, haplotyping and association studies, identify candidate regions containing a disease susceptibility gene by exploiting the cosegregation of the disease phenotype and markers in cases as well as the difference of allele sharing between cases and controls. The determination of haplotype sharing may represent the future direction of linkage analysis due to its better adaptation to high-density SNP genotype data [2]. Tools that can make efficient use of the profound information from high density SNP genotype become increasingly important.

It is currently still difficult to obtain haplotype information on a genome wide basis for high-density SNP genotype data from either a general pedigree or a population. Performance of haplotype inference methods in a general pedigree relies on whether enough informative individuals have been genotyped. Inference of haplotypes from a diploid population is a NP-hard problem [3]. Many methods have been developed to reconstruct haplotypes, including parsimony approaches [3], [4], maximum-likelihood methods [5]–[8], phylogeny-based approaches [9], [10], and other statistical methods [11], [12]. Such algorithms have been implemented in widely used linkage packages including Vitesse [13], GeneHunter [14] and Simwalk2 [15]. However, for very large pedigrees combined with large marker genotype data sets, analytical approaches quickly become computationally intractable, and even sampling methods such as Simwalk2 are computationally slow and hardware-intensive. Thus the utility of likelihood based approaches is still limited.

The non-parametric Homozygosity Haplotype (HH) method (HH is not homozygosity mapping employed to search for segments inherited homozygous by descent in inbred recessive pedigrees) was proposed by Miyazawa *et al*. [16] recently for a genome-wide search of shared autosomal segments with high density SNP genotype data for families or genetically isolated populations. Rather than formally phasing haplotypes, the HH approach utilizes a reduced haplotype described by the homozygous SNPs only. Homozygosity haplotype is easily obtained by removing all heterozygous SNPs from a sample data set. The reduced haplotype of each chromosome is then uniquely phased by the string of the remaining homozygous SNPs. For patients who inherited the same mutation from a common ancestor, they share a chromosomal segment identical by descent (IBD) around the disease susceptibility gene. For both autosomal dominant and recessive inheritance, patients should not have discordant homozygous calls in the IBD. In HH, they share the homozygosity haplotype in the IBD interval. An IBD segment is denoted as a region from a common ancestor (RCA) in the HH approach. RCA is identified by comparing the homozygosity haplotypes among patients. The conventional haplotype analysis of phasing diploid alleles into haploid alleles is greatly simplified by the idea of homozygosity haplotype, which allows the HH program to perform genome-wide analyses in minutes. However, the practical performance and utility of the HH method have not been extensively examined.

In this study, the applicability and the effectiveness of the HH approach in localizing causative genes are presented and discussed for a series of monogenetic disease projects with high-density SNP genotype data. A large Canadian family with Schnyder crystalline corneal dystrophy (SCCD, MIM 121800) for which the disease susceptibility gene was discovered recently [17]–[19], was used to validate the HH approach. We further tested the HH method for identifying potential linkage to known genes in novel families or cohorts. When a genetic disease is diagnosed, screening known causative genes is an important procedure to provide further clinical service. As one often finds when searching a phenotype in the Online Mendelian Inheritance in Man (OMIM) database [20], a disease phenotype is often linked to multiple genes or loci. Sequencing and analyzing known genes is still time-consuming, especially when the detected variants have not been published before. Microsatellite genotyping is often used to detect linkage of known loci. Genotyping with microsatellites are more labor intensive and require more detailed analysis in calling the genotypes. Assuming that patients who have inherited a susceptibility gene from a common ancestor also share a haplotype in the genomic interval, the HH approach can be applied to screen the known causative genes or loci by searching for the shared homozygosity haplotype. We show that HH mapping correctly identifies the causal locus for a retinal degenerative disorder, and excludes linkage to known genes for a cohort with a novel renal failure phenotype.

## Results

### Validation of HH using a large family with SCCD

**Figure 1:**
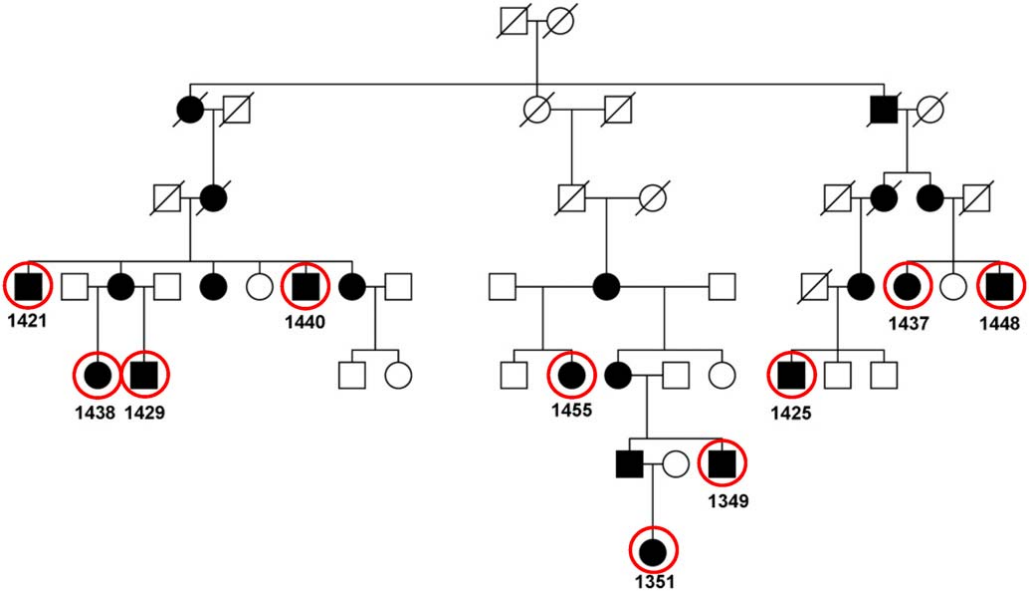
Pedigree of a family with Schnyder crystalline corneal dystrophy. Genotyped subjects are 1351, 1349, 1429, 1438, 1455, 1425, 1421, 1440, 1448, and 1437.

Schnyder crystalline corneal dystrophy (SCCD, MIM 121800) is a rare autosomal dominant disease characterized by progressive opacification of the cornea resulting from the local accumulation of lipids, and associated in some cases with systemic dyslipidemia. A large multigenerational family (Fig. 1) in Nova Scotia was ascertained with SCCD. Previously published genetic analyses in other families suggested linkage of SCCD to an interval on chromosome 1. Intensive microsatellite fine mapping on 37 informative members of our family confirmed linkage to this interval, refining it to 1.3 Mbp. The underlying causative gene, UBIAD1, was identified by sequencing genes of the interval [17]. We utilized the SCCD family to test whether the HH algorithm could correctly identify the causal locus.

**Figure 2:**
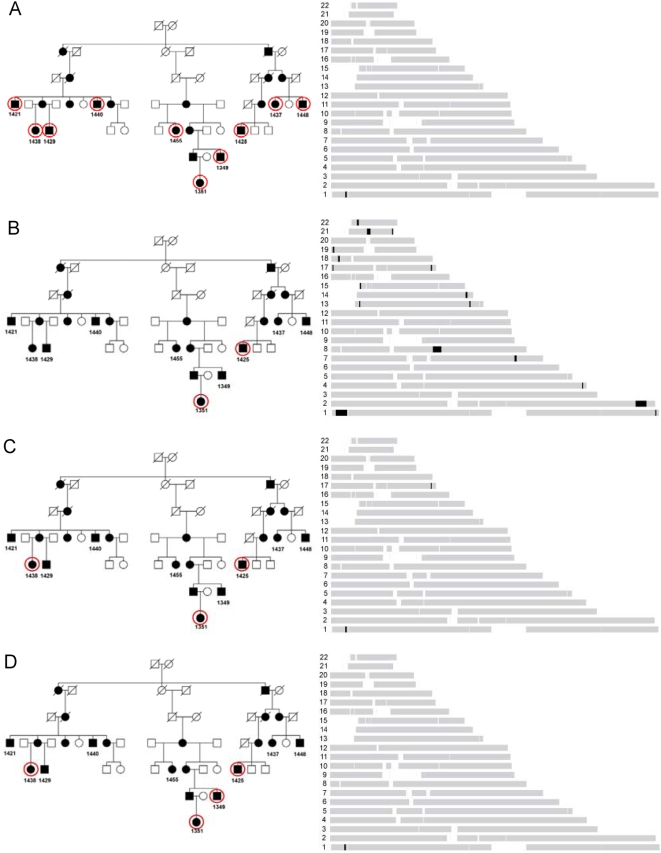
Identification of the candidate regions for the family with Schnyder crystalline corneal dystrophy using HH approach. (a) RCHHs shared by 10 patients. The RCHH intervals are shown in black. Other autosomal regions are shown in grey as background. (b) RCHHs shared by 1351 and 1425. (c) RCHHs shared by 1351, 1425, and 1438. (d) RCHH shared by 1351, 1425, 1438, and 1349.

Samples from 10 affected individuals were genotyped with Illumina HumanHap550 chips. The HH program was first run with the genotypes of all 10 patients with a cutoff value of 3.0 cM, which is the recommended cutoff of HH approach [16]. Within one minute, HH analysis identified a single RCHH (Region with conserved HH) on chr1: 10,686,402–11,639,887 bp with a total of 175 informative SNPs and an interval size of 953,486 bp (Fig. 2a). This chromosomal region correctly includes the causative gene UBIAD1. The defined interval is smaller than the 1.3 Mbp interval identified previously by fine mapping with customized microsatellites [17], or the 2.32 Mbp region reported by Theendakara *et al.* [21], indicating superior resolution of the dense SNP marker panel in this instance.

We next sought to determine whether it was necessary to include all 10 patients in order to identify the correct locus in this pedigree. According to equation (1), the ratio of RCA shared by patients decreases with the number of generations removed from their common ancestor. Therefore, samples from the youngest generation of different family branches should be more informative in general. By selecting samples according to this rule, subject 1351 and 1425 (*m*+*n* = 10) were selected first, and the HH analysis result is shown in Figure 2(b). Patients 1351 and 1425 shared a few other large RCHHs in addition to the largest interval at chr1: 3,539,057–11,973,221 with a size of 8.4 Mbp. Thus two affected subjects were insufficient to determine a unique genomic location of the causal locus. By next adding most distantly related individual, 1438, only two RCHHs were left (Figure 2(c)), and the largest RCHH on chromosome 1 decreased to 0.9 Mbp. The second, incorrect RCHH on chromosome 17 could be excluded by adding any one genotype of other affected individuals. As shown in Figure 2(d), the only RCHH shared by the four individuals 1351, 1425, 1438, and 1349 is the same interval as the one identified using 10 samples. This study of sample subsets demonstrates that the optimal genotype data set for HH analysis should be those from the youngest generation or otherwise most distantly related affected individuals in a pedigree.

### Application of HH to identify the disease linked region for a family with retinal degeneration

Although affected individuals are the most informative for non-parametric analysis, the statistical functions of HH (see equation (2) and (3)), which were developed to study multigene diseases using genotypes of both patients and controls, can be extended to include unaffected family members. This is similar in spirit to a family-based association approaches [22]–[24]. In general, the test statistics for association analysis may be regarded as a test for the presence of a difference in allele frequency between cases and controls. The HH algorithm can be adapted to treat affected and unaffected members of a family as cases and controls respectively.

**Figure 3:**
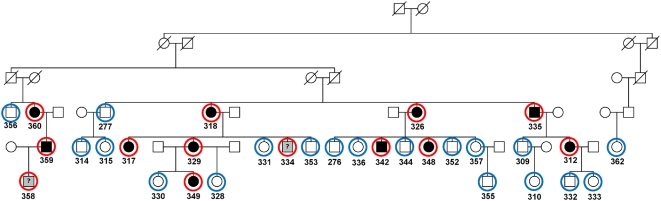
The pedigree of a family with a progressive retinal degenerative disorder. Genotyped individuals are indicated with red circles (affected) and blue circles (unaffected).

**Figure 4:**
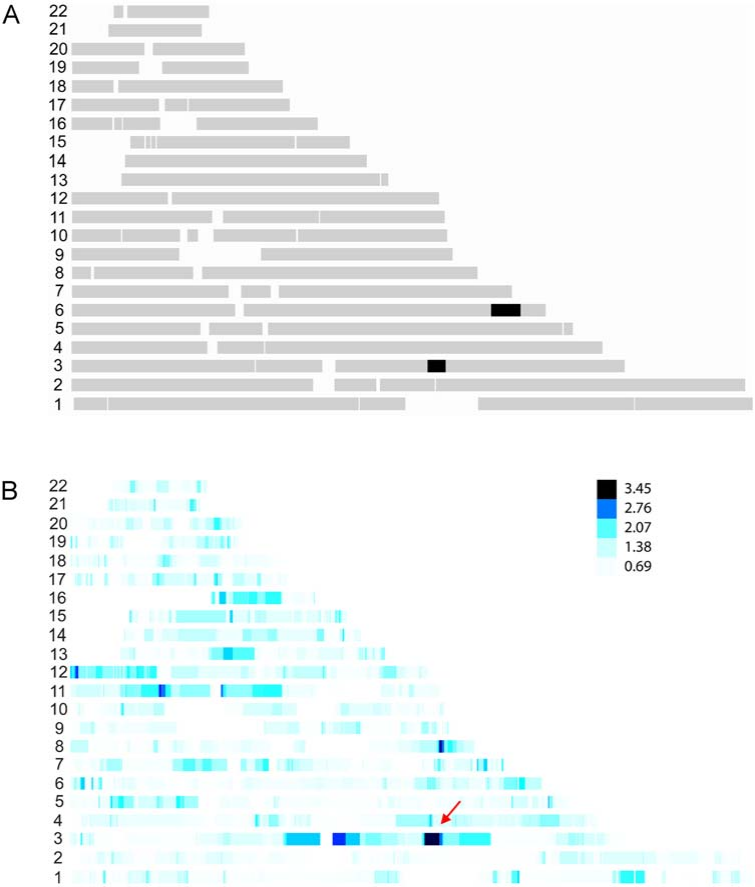
Using HH method to identify the candidate regions with both cases and controls for the family with progressive retinal degenerative disorders. (a) RCHH mapping using the 300K SNP genotypes of eight affected individuals 312, 317, 318, 326, 329, 335, 349, and 360. The RCHH intervals are shown in black. Other parts of autosomes are shown in grey as background. (b) Densitogram of −log_10_^(P)^ value for the representative RCHHs shared by patients with the unaffected individuals as controls. The darker the color, the more significant the RCHH is. HH analysis was run using affected family members as cases, unaffected family members as controls. The subjects used to build the patient pool were 312, 317, 318, 326, 329, 334, 335, 342, 348, 349, 358, 359, and 360, in which 334 and 358 were subsequently re-diagnosed as suspicious unaffected. Samples in control pool were 276, 277, 309, 310, 314, 315, 328, 330, 331, 332, 333, 336, 344, 352, 353, 355, 356, 357, and 362.

**Figure 5:**
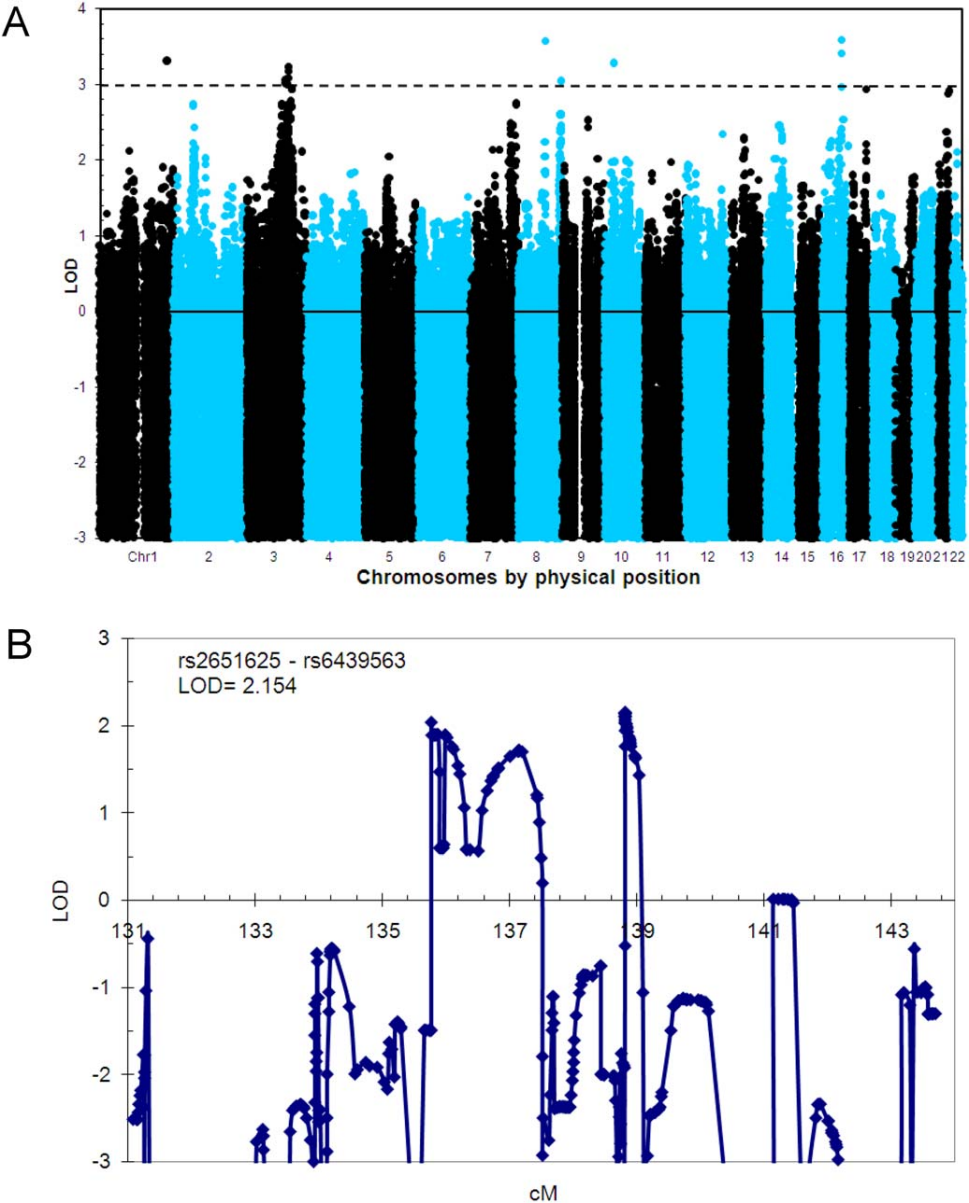
Linkage analysis results for the family with progressive retinal degenerative disorders. (a) Whole genome scan 2-point linkage result. (b) Multipoint linkage result on chromosome 3.

We ascertained a large Canadian family with a specific pericentral retinal disorder (PRD) (Fig. 3). Thirty-four family members were genotyped with the Illumina HapMap300 beadchip with a total of 318,237 SNPs. We first performed HH analysis using genotypes of eight definitively affected family members only, with a pairwise cutoff value of 3.0 cM to search the candidate RCHHs. As shown in Fig. 4a, the affected-only analysis identified two large RCHH regions. One was on chromosome 3 at 128–134 Mbp, the other on chromosome 6 at 151–161 Mbp. We then performed the HH statistical analysis using both the patient pool and the control pool (Fig. 4b). Of the two RCHH regions previously defined by affecteds only, the physically larger region on chromosome 6 lost significance. Whereas the region at chr3: 128,295,267–133,701,313 became the most significant candidate locus with the largest −log_10_ *^P^* value of 3.35. The region was confirmed later by the whole genome scan results of two point and multipoint linkage analyses. Two point linkage analysis was carried out using the MLINK routine of FASTLINK v4.1P [25], [26] with a dominant transmission model: Penetrance of 0.95, phenocopy of 0.001, and the disease allele frequency of 0.001. Two point linkage identified 6 regions with LOD scores >3.0 (Fig. 5a). The extended interval on chromosome 3 was the most consistent one. Multipoint linkage analysis was carried out using SIMWALK2SNP [15], [27], [28] version 2.91 with the same dominant transmission model. Tag SNPs were selected from the 6 regions using Haploview [29] for multipoint linkage analysis. The chromosome 3 interval was identified with the highest LOD score 2.15 of all tested intervals (Fig. 5b). The region encompasses the gene encoding rhodopsin. A missense mutation in rhodopsin was detected by direct resequencing as the presumptive causative mutation in the pedigree. These results demonstrate that the HH approach can be effectively applied to study monogenic traits in large families by utilizing the genotypes of both affected and unaffected family members.

### Application to the screening of known causative genes

**Figure 6:**
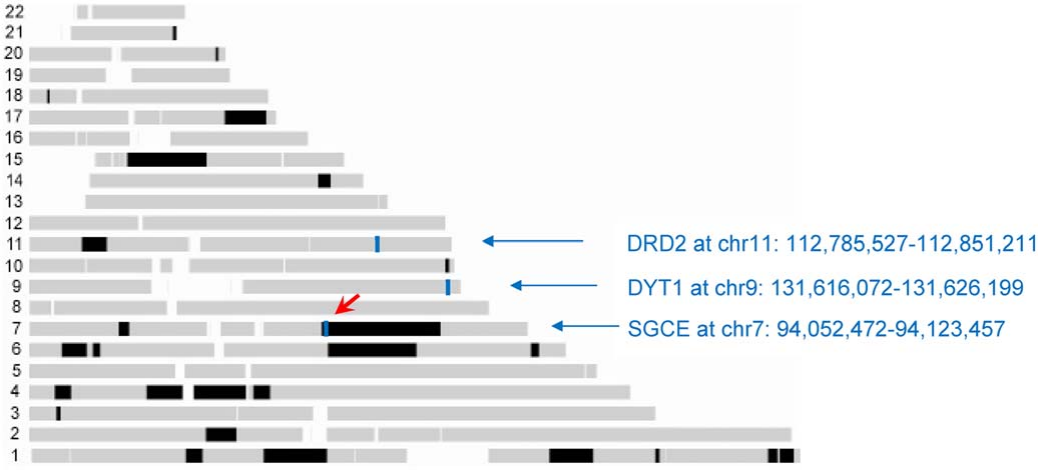
Screening of the three known causative genes DRD2, DYT1, and SGCE for a Canadian family with myoclonus dystonia using HH approach and the genotypes of four patients.

**Figure 7:**
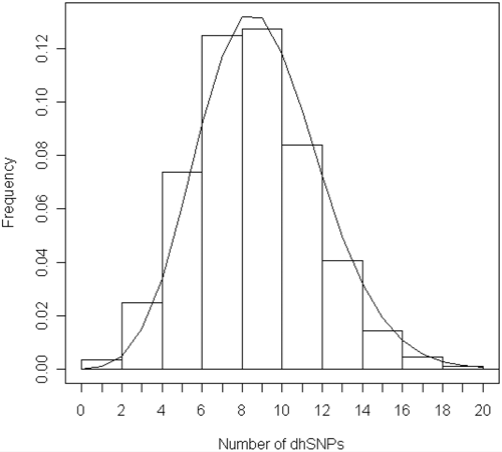
Histogram distribution of the dhSNPs introduced by genotyping error. Error simulation was performed on the genotype data of Myoclonus dystonia patients in region Chr11:111,851,211–113,785,527 including DRD2 gene. The curve is the fitted Poisson distribution curve with λ = 8.98 (σ = 0.03). Genotyping error was simulated using error model 1 with an error ratio 0.01. Monte Carlo error simulation was run 10,000 times.

The whole-genome screening approach was further validated using a Canadian family ascertained with myoclonus dystonia (MIM 159900). Three causative genes are known for this genetic condition: SGCE [30], DRD2 [31], and DYT1 [32]. Direct sequencing concurrent with our analysis identified a null mutation in the SGCE gene in affecteds from this family. We tested whether the HH approach could exclude non-causative genes correctly. Four patients from the family were genotyped with the Illumina HumanHap550 beadchips. HH was run first to identify RCHHs shared among the four patients with a cutoff of 3.0 cM. The RCHHs are given in Table 1, and the genome-wide mapping of RCHHs is shown in Fig. 6. The largest RCHH at chr7:93,168,493–130,965,632 with size of 37 Mb includes gene SGCE (chr7:94,052,472–94,123,457). No RCHH was found around the DRD2 (chr11:112,785,527–112,851,211) or DYT1 (chr9:131,616,072–131,626,199) genes. The genotypes of the genomic regions with DRD2 and DYT1 inside were further analyzed to examine the effect of genotyping errors on their exclusion. The genotyping error simulation method (see Methods) was applied to calculate the reliability of genotype data, the results are shown in Table 2. The discordant homozygous SNPs (dhSNPs) of the original genotypes in the regions were permutated to concordant SNPs. MC simulation was run 10,000 times on the transformed genotype data with both error model 1 and 2 and a high error ratio of 0.01. The distributions of the number of dhSNPs produced by the simulated genotyping errors were then fitted with a Poisson distribution (as the example shown in Fig. 7). Based on the obtained distribution, the P values are all approximately 0. The simulation results suggest that the dhSNPs in the original genotype used to define RCHH are reliable. Consequently, the DRD2 and DYT1 genes were excluded with high confidence. Thus the HH approach correctly interpreted unlinked candidate genes, and identified the potential linkage of SGCE in the meanwhile.

**Table 1:** List of RCHHs shared by the four patients from a Canadian family ascertained with myoclonus dystonia.

SNPs	Chromsome	Start(SNP)	Start(bp)	End(SNP)	End(bp)	Size(bp)
6193	7	rs10243929	93,168,493	rs929731	130,965,632	37,797,140
5156	15	rs12443212	31,309,577	rs11855284	56,476,479	25,166,903
4987	6	rs6914928	95,181,973	rs9398707	123,268,276	28,086,304
3814	1	rs17095322	74,676,941	rs957334	94,801,569	20,124,629
3041	17	rs17644943	62,338,082	rs1622986	75,438,157	13,100,076
2677	4	rs1910739	52,378,364	rs17652284	68,905,652	16,527,289
2534	1	rs12095738	165,746,611	rs2609473	179,602,130	13,855,520
2063	4	rs2911902	37,467,034	rs1051447	48,758,629	11,291,596
1977	11	rs11024074	16,873,795	rs4465366	24,639,693	7,765,899
1915	6	rs7753334	10,403,729	rs214582	18,324,763	7,921,035
1668	2	rs7576924	56,355,325	rs3919602	65,890,044	9,534,720
1234	14	rs4900132	92,029,991	rs8019939	95,934,062	3,904,072
1044	4	rs1878519	8,161,682	rs7688193	13,344,993	5,183,312
962	1	rs2995381	239,115,984	rs6656693	243,402,266	4,286,283
916	7	rs160346	28,532,166	rs917749	31,761,680	3,229,515
786	1	rs10925300	235,283,523	rs1982530	238,365,440	3,081,918
738	1	rs2051086	49,877,788	rs1469344	55,158,755	5,280,968
708	4	rs4694317	71,386,025	rs4859537	76,745,685	5,359,661
668	6	rs10945617	159,872,422	rs1790004	162,302,229	2,429,808
546	6	rs2148943	20,254,160	rs1047953	22,294,843	2,040,684
359	1	rs3767514	199,383,756	rs2741853	200,672,294	1,288,539
346	3	rs11131140	8,650,778	rs2479	9,883,525	1,232,748
327	10	rs11017516	132,538,433	rs7084312	133,703,278	1,164,846
235	20	rs1892318	59,421,464	rs6089695	60,241,421	819,958
204	21	rs914238	45,840,089	rs9979962	46,792,735	952,647
180	18	rs6506336	5,833,069	rs11873891	6,495,597	662,529
62	6	rs1891086	22,330,108	rs4560628	22,559,799	229,692

**Table 2:** The error possibilities calculated using genotyping error simulation method in the screening of the known causative genes for a family with myoclonus dystonia with the genotype data of four affected individuals.

Gene	Region	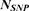	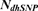	Error Model1	Error Model2
				E = 0.01	t = 0.005
DRD2	Chr11:111,851,211–113,785,527	493	77	λ = 8.98	λ = 4.24
				P = 0	P = 0
DYT1	Chr9:130,626,199–132,616,072	480	72	λ = 8.76	λ = 5.05
				P = 0	P = 0

**Figure 8:**
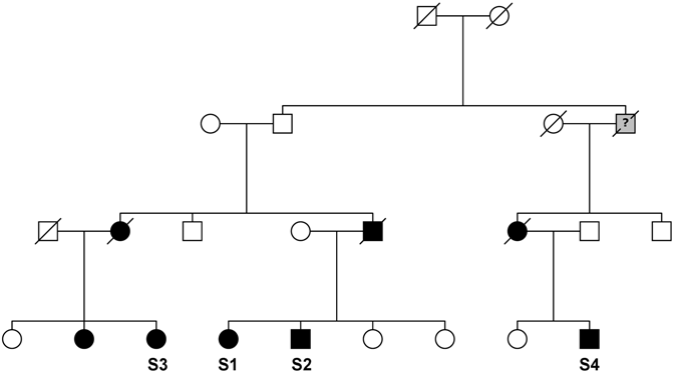
Pedigree of a family with renal failure.

The selection of the cutoff value and samples is critical in employing the HH method. We used a family we ascertained with generalized renal failure as an example to illustrate the selection procedure. Four patients S1, S2, S3, and S4 from the family (Fig. 8) were genotyped with Illumina HumanHap550k beadchips. We tested for linkage of the family to either of two known causative genes for related renal conditions: PKD1 (chr16:2,078,712–2,125,900) and PKD2 (chr4: 89,147,844–89,217,952). HH analyses of the four affected samples taken together showed they do not share RCHH covering the two genes.

The genotyping error simulation method was applied to study the error possibilities using 550k genotype data of two affected subject s1 and s4. The Monte Carlo simulation of genotyping error was run 10,000 times for each test. As shown in Table 3, the P values are all lower than 0.001. As a consequence, PKD1 and PKD2 could be confidently excluded as causal for the phenotype in this family, which is now undergoing further mapping to identify a linked causal locus. To demonstrate the selection of cutoff value and samples, the results of HH analyses with different subsets of the four genotyped subjects and different RCHH cutoff values are shown in Table 4. PKD1 and PKD2 could not be excluded when using closely related sample subsets, e.g. S1-S2 (*m* = 1, *n* = 1), or S2-S3 (*m* = 2, *n* = 2). However, all subsets with more distantly related patients, including S2-S4 (*m* = 3, *n* = 3) and S3-S4 (*m* = 3, *n* = 3), were successful in ruling out the PKD1 and PKD2 genes as compared to the full analysis using all four samples. The results show that the selection of more distantly related samples would give a higher success rate for the screening of known causative genes. In addition, the performance of screening can be improved by adding more genotype data of affected individuals. For cutoff value selection, the HH analyses with a small cutoff of 1.0 cM were not able to exclude PKD1 and PKD2 when the patients were more closely related, e.g. subset S1-S2, S2-S3 and S1-S3. For the two descendants / patients from a family with 2≤*m*+*n*≤6, a condition suitable for a general family, a cutoff of 2.0 cM is recommended for the screening of known causative genes because the ratio of type II errors in identifying candidate region to the total length of the RCAs is lower than 0.01 [16]. When the subjects are too closely related, *e.g.* an affected sib pair with *m*+*n* = 2, it is suggested to genotype more affected individuals.

**Table 3:** The error possibilities calculated using genotyping error simulation method in screening known causative genes PKD1 and PKD2 for a family with renal failure with the genotype data of patient s1 and s4.

Gene	Region	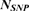	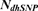	Error Model1	Error Model2
				E = 0.01	t = 0.005
PKD1	Chr16:1,125,900–3,078,712	277	9	λ = 1.72	λ = 0.79
				P = 7.73e–5	P = 1.60e–7
PKD2	Chr4:88,217,952–90,147,844	388	15	λ = 2.47	λ = 0.98
				P = 5.81e–8	P = 2.17e–13

**Table 4:** The effect of using subsets of the affected individuals (s1, s2, s3 and s4) and different cutoff values in the screening of the known causative genes PKD1 and PKD2 for a family with renal failure.

Samples	Cutoff 1.0 cM		Cutoff 2.0 cM		Cutoff 3.0 cM	
	PKD1	PKD2	PKD1	PKD2	PKD1	PKD2
s1, s2; *m* = 1, *n* = 1	−	−	+	−	+	−
s2, s3; *m* = 2, *n* = 2	−	+	−	+	+	+
s1, s3; *m* = 2, *n* = 2	−	−	+	+	+	+
s1, s4; *m* = 3, *n* = 3	−	+	+	+	+	+
s2, s4; *m* = 3, *n* = 3	+	+	+	+	+	+
s3, s4; *m* = 3, *n* = 3	+	+	+	+	+	+
s1, s2, s3	−	+	+	+	+	+
s1, s2, s4	+	+	+	+	+	+
s2, s3, s4	+	+	+	+	+	+
s1, s3, s4	+	+	+	+	+	+
s1, s2, s3, s4	+	+	+	+	+	+

‘+’ indicates no RCHH shared by patients is found flanking the gene, and suggests the gene is excludable; ‘−’ indicates an RCHH is found flanking the gene. *m* and *n* are the number of generations of the two patients descended from their common ancestor.

### Impact of SNP genotyping errors on HH analysis

As other statistical genetics methods, the accuracy of HH approach is also affected by genotyping errors. Genotype errors may impact HH results in two ways. Genotype errors may break down a large RCHH to a few smaller RCHHs or intervals, and make the RCHH undetectable if the intervals are smaller than the cutoff value. Second, genotype errors may create a false RCHH. An interval with discordant homozygous SNPs can be identified as a false RCHH if all the discordant SNPs are changed to concordant SNPs by genotyping errors.

For the first possible impact, a genotype error must change a non-dhSNP (either a non-comparable SNP or a matched comparable SNP) to a dhSNP (a mismatched comparable SNP). For a non-dhSNP, when at least one sample is homozygous for the SNP, there is a half chance to cause an error if an AB call was changed to AA or BB. Whereas the change of a homozygous call AA or BB to AB has no effect, the changing to the opposite homozygous call will cause an error only if other homozygous calls of the tested samples are also present. Given an error ratio *ε*, the chance of generating a false dhSNP by genotype error is ≤*ε*/2. For the second potential impact, genotype errors may create a false RCHH by changing all dhSNPs of an interval to non-dhSNPs. Given an error ratio of *ε*, an interval with total 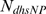 of dhSNPs for two samples, the chance of creating a false RCHH by genotype errors is 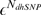. The possibility would be lower when using multiple samples because either all AA or all BB calls should be changed by genotyping error. The possibility of generating false RCHH by genotype errors is apparently very low. Therefore, the major impact of genotype errors on HH analysis is to break down a large RCHH.

## Discussion

In this study, we investigated the applicability and the effectiveness of HH in identifying genetic disease genes with a series of monogenetic disorders ascertained in eastern Canadian populations. We applied HH to identify the known locus using the genotypes of 10 affected subjects of a family with a rare dominant eye disease SCCD. HH analysis successfully detected the 1 Mb shared segment of the affected members with a minimum of four samples. This study of sample subsets also demonstrates that the optimal genotype data set for HH analysis should be those from the youngest generation or otherwise most distantly related affected individuals in a pedigree. This suggests that important upstream efficiencies can be realized in the clinical phase of genetic studies by the adoption of a sparse recruitment strategy focused on the most informative individuals. We also explored the applicability of HH to the family based case and control study. Thirty-four family members from a large Canadian family with a specific retinal disorder PRD were genotyped with the Illumina HapMap300 beadchip. With 13 affected samples in the patient pool and 19 samples in the control pool, HH identified an RCHH on chr3 with the lowest *P* value. The interval was confirmed by the two point and multipoint linkage analyses, and for which the causative mutation was found by direct resequencing. Clearly the HH approach can be effectively applied to study monogenic traits in large families by utilizing the genotypes of both affected and unaffected family members. HH can also be applied to test known causative genes or loci quickly for potential linkage. The whole-genome screening approach was further validated using a Canadian family ascertained with myoclonus dystonia and a renal failure cohort. HH correctly detected the potential linkage of myoclonus dystonia, and excluded the known causative genes for the renal failure cohort. These applications demonstrate that HH is an efficient and reliable tool in identifying the potential linkage for monogenic diseases. In addition, the successful application of HH to our projects indicates that HH is well suited for founder populations like those in Atlantic Canada. The population is considered a geographically isolated population derived from thousands of ancestors with *m* = *n* = 20.

The HH approach is very efficient and easy to implement. In comparison to other linkage analysis programs, the most significant advantage of HH is its high computational efficiency. The time complexity of HH algorithm is *O*(*n^2^*). It was reported that the calculation for a family with Marfan syndrome that contains nine subjects is completed in 6 s on a laptop, and another analysis composed of two pools containing 45 subjects each took 5 min [16]. All the HH analyses performed in this study were finished in less than one minute on a computer station with Intel Xeon 3.2 GHz processor. HH analysis can be run on personal computers. Additionally, HH is easy to implement and use. HH can be run on Mac with graphical user interface. Most of the work in implementation involves generating the proper input genotype data format and the annotation file for a particular SNP chip. HH is a non-parametric method. Hence there is no need to specify a complete genetic model. When HH is applied to study a large family affected with a genetic disease, it is usually not necessary to genotype many family members because more distantly related subjects are theoretically more informative in HH analysis. One may start by genotyping several affected subjects from the youngest generation, subsequently narrowing the RCHH down by adding more subjects to the analysis as required.

One feature of the HH method that can be improved is the usage of cutoff value. In our applications, we found it difficult to determine an optimal cutoff value. A cutoff value of 3.0 cM was used by the Miyazawa *et al.* [16]. In general, there is a tradeoff between the size of the cutoff value and the ability to correctly define a single truly linked locus. Miyazawa *et al.* [16] have discussed extensively the relationship of cutoff values and the type I and type II errors in identifying candidate regions. The average genetic length of the RCAs decreases over generations. Therefore, more distantly related subjects tend to share smaller RCA and a smaller cutoff should be used. One possible solution is running the analyses by gradually reducing the cutoff value until an RCHH appears. Sometimes a cutoff value 3.0 cM will not identify any RCHH. On the other hand, the results obtained using a smaller cuttoff value of 1.0 cM can have many undistinguishable false positives. The problem can be partly solved if a significant RCHH can be identified by adding unaffected subjects as controls. In case no controls can be used, it is an issue if there are multiple RCHHs without significant differences in size.

In this study, we analyzed the impact of genotype errors on HH analysis. The major impact of genotype errors on HH analysis is breaking down a large RCHH. Several approaches may be useful to reduce the impact of genotype errors. Miyazawa et al. [16] used a confidence value cutoff to exclude genotype calls with low confidence. Checking Mendelian inconsistencies can remove some of the errors if the genotyped samples are suitable for running a Mendelian test. However, 100% of errors can not be excluded. Hao *et al.* [33] studied the genotype errors of GeneChip Mapping 10k array, and found that the average genotyping error rate of this SNP genotyping technology was about 0.1%. Illumina reported that the rate of Mendelian inconsistencies of Illumina HumanHap550 beadchip is 0.06% [34]. Genotyping errors may or may not cause Mendelian inheritance incompatibilities, SNP genotyping error-detection rates have been studied using trio designs, and it has been estimated that Mendelian incompability errors in theory may detect no more than 61% of all possible errors [35], [36]. The difference between the true and the estimated error rates is mainly due to errors that are ‘Mendelian compatible’ [37]. As a consequence, it is estimated that the average genotyping error rate of the 550k SNP genotyping is close to 0.1%. But the actual error rate usually varies for different batch of data. The error simulation method presented in this study, in which a high error ratio of 1.0% was selected to simulate the effect of potential genotyping errors under the worst situation for the purpose of screening known genes, could be an alternative solution.

### Conclusions

Our study of the HH approach with Illumina high-density SNP genotype data from a series of Atlantic Canadian monogenetic disease projects demonstrates that HH is very efficient and effective in identifying disease linked and unlinked regions. The method can be used as an efficient alternative approach to sequencing or microsatellite-based fine mapping for the research and clinical diagnosis of genetic diseases.

## Methods

### Important concepts of HH method

#### HH

An HH is a haplotype described by only homozygous SNPs. It is obtained by deleting heterozygous SNPs. Then, the haplotype of each chromosome is uniquely determined by the combination of the allelic type in each homozygous genotype.

#### Comparable SNP

A comparable SNP is a SNP that is homozygous in two subjects. HH can be compared between two subjects using the comparable SNPs. The mismatched comparable SNP has discordant homozygous SNP genotypes in two subjects, one is AA and the other is BB. We used dhSNP (discordant homozygous SNP) to represent the mismatched comparable SNP.

#### Region with conserved HH (RCHH)

RCHH is a run of matched comparable SNPs with genetic length longer than a cutoff value. A RCHH is bounded by either dhSNPs or by the ends of the chromosome. HH approach searches RCHHs between each pair of subjects. The overlapped RCHH shared by multiple subjects is used to predict the presence of region of conserved ancestry (RCA) or identity by descent (IBD).

#### RCA sharing

For the two descendants with *m* and *n* generations removed from a common ancestor, the ratio of the total genetic length of the derived RCA to the entire length of the autosomes is denoted as *RCA(m,n)*, which can be calculated with
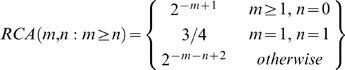
(1)


#### Statistics in using patient and control pools

The HH program also has a statistical method developed to identify candidate regions for multigene diseases in genetically isolated populations by comparing the shared RCHHs between the patient pool and the control pool [16]. In the algorithm, an autosomal interval is firstly divided into minute regions. The RCHH shared by the largest number of patients in the patient pool is then selected as the representative RCHH for each small region. After that, the numbers of subjects who share the representative RCHH were counted for both the patient pool and the control pool. Finally, the significance of each representative RCHH is calculated. The numbers of subjects who share an RCHH at a given position on an autosome were compared between the patient pool and the control pool. The assumption was made that
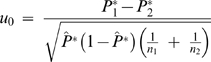
(2)has a standard normal distribution, where 
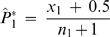
, 
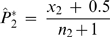
, 
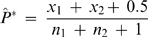
, *x*_1_ and *x*_2_ are the numbers of subjects sharing RCHHs in the patient pool and the control pool, respectively, and *n_1_* and *n_2_* are the total numbers of subjects in the patient pool and the control pool, respectively. The significance of each representative RCHH is expressed with the *P* value, which is calculated by 
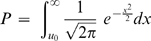
(3)


### Genotyping

Samples were genotyped with the Illumina HumanHap300K or HumanHap550K SNP genotyping beadchips. The Illumina HumanHap300 chip has a total of 318,237 SNPs, and the HumanHap550K genotype has a total of 561,466 SNPs. Genotype data of subjects were transformed to the input format of HH program.

### Implementation of HH

The source code of HH program [16] was obtained from the author. The source code was modified to customize the output format of the RCHH list. The revised C source code of the HH program was compiled with GNU compiler on a Linux-based operating system Fedora. The parameter LARGEGAP defined in the header file, which is used to define large gaps like centromere, was changed from the default value of 300,000 bp to 400,000 bp to accommodate some non-centromere spaces between two consecutive SNPs. The SNP annotation file provided by HH software is for the Affymetrix 500K GeneChips Human Mapping Array Set. Annotations of SNPs include fields of SNP name, physical coordinates, genetic distances, and minor allele frequencies. The annotation files for Illumina HumanHap300K and HumanHap550K SNP genotype were created according to the requirement of the HH program with the SNP annotations downloaded from Illumina technical support ftp site. The genetic distances of SNPs with empty value, inconsistent value, or zero were interpolated according to the physical coordinates of their flanking SNPs. The SNP annotations for CEU population were used in this study.

### Method of genotyping error simulation for the screening of known causative genes

#### Genotyping Error Model

Two commonly used error models were implemented. Genotyping error was introduced randomly in the SNP genotypes of each individual with a given error rate.

#### Model 1

The model was first introduced by Lincoln and Lander [38], which is widely used [39]–[41]. The model assumes a uniform distribution of errors over the available genotypes at a locus. For SNP genotype, the penetrance function of genotyping error is 
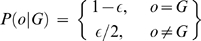
In which, *o* is the observed genotype of a SNP, *G* is the underlying genotype, the genotype error rate is *ε*. The error rate is the same for all possible underlying genotypes.

#### Model 2

This model uses mean error rate per allele to quantify genotype errors [33], [35], [37]. In the random error model, it is assumed that the average probability of misclassifying allele A or B to B or A is equal, which can be denoted as 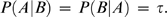
In which, *τ* is the allelotyping error rate. Thus, the penetrance of error genotype follows
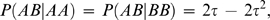


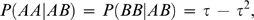
and
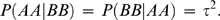

*τ* can be approximated to *ε*/2 when *τ* is small [33].

#### Statistics of genotyping error

An RCHH will be broken down if genotyping errors change non-dhSNPs to dhSNPs. Furthermore, the RCHH will not be identified if the resulted segments are smaller than the cutoff in genetic distance. As a result, the causative gene will be excluded mistakenly because of the impact of genotyping error. To investigate the possibility of this type of error, Monte Carlo (MC) simulation was used to simulate the influence of genotyping error.

First, the genotype of all tested samples in the region with a known causative gene was modified by replacing the dhSNPs with non-dhSNPs; thus the modified genotypes had consistent homozygosity haplotype. Given a gene/loci at chrN with start position at P1(bp) and stop position at P2 (bp), the region with a known gene/loci is defined as on chrN, starting at P2-1.0 Mb, and ending at P1+1.0 Mb. Then, the MC simulation was performed on the modified genotypes with the selected genotyping error model and error rate. After a large number of runs of MC simulation on each individual's genotype, the distribution of the number of dhSNPs created by genotyping errors was analyzed using the Poisson distribution with the probability density function in equation (4). The Poisson distribution is discrete, and has only a single parameter λ that is both the mean and the variance.
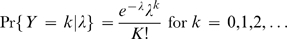
(4)The possibility of the observed dhSNPs generated by genotyping error was calculated according to equation (5). The P value, the possibility of getting 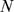 number of dhSNPs generated by genotyping error in a region without dhSNPs is
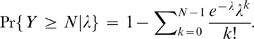
(5)


The error simulation results, *i.e.* the number of dhSNPs for each run, can be imported to R [42] to calculate the possibility of getting 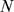 number of dhSNPs by genotyping error. If the possibility is very low, *e.g.* P<0.001, that is, the dhSNPs in the region are not likely created by genotyping error. Consequently, the genotype and HH results are reliable.
